# Supplementary material for: Changes in Dietary Patterns through a Nutritional Intervention with a Traditional Atlantic Diet: The Galiat Randomized Controlled Trial
Source: Nutrients. 2021 Nov 25;13(12):4233. doi: 10.3390/nu13124233 (PMC8704078; doi:10.3390/nu13124233)
Supplement: Supplementary file 1 [file nutrients-13-04233-s001.zip › Supplementary Table S1.pdf]

Supplementary Table S1. Food groupings used in the dietary pattern analysis.

Food groups	Foods items
Low-fat dairy products	Skimmed and semi-skimmed milk, yogurt or kefir, with added sugar or not; skimmed and semi-skimmed cheese
Whole fat dairy products	Whole milk, whole yogurt or kefir; fatty cheese; butter; fermented milk beverages; sugary milk derivatives such as milk cream, milk shakes
Fried or scrambled eggs	Fried or scrambled eggs
Boiled or poached eggs	Boiled or poached eggs
Cooked, steamed, roasted meats	Chops, steak, bovine, pork, poultry, etc.
Fried meats	Chops, steak, bovine, pork, poultry, etc.
Processed meats	Hamburgers, sliced and preserved ready-to-cook products: sausages, ham, loin
Boiled fishes and seafood	Boiled fishes and seafood
Fried fishes	Fried fish and fish fingers
Vegetables	Raw vegetables, usually mixed in a salad (e.g., cucumber, lettuce, tomato); cooked vegetables (e.g., cabbage, broccoli, green bean)
Boiled potatoes	Boiled potatoes
Fried potatoes	Fried potatoes, chips
Fruits	Fresh fruit (also freshly made fruit smoothies)
Legumes	Legumes (lentils, chickpeas, beans)
Sweets	Snacks like biscuits, packaged cakes, pastries, puddings, cereal bars, candies, loose candies, marshmallows, ice cream, milk or fruit-based bars, chocolate, candy bars
Honey	Honey
Whole grains	Whole grain bread, dark roll, dark crispbread, whole meal pasta, brown rice
Refined grains	White bread, white roll, white crispbread, breakfast cereals, pasta, noodles, white rice
Pizza	Pizza
Nuts	Nuts, seeds, dried fruit
Salty snacks	Snacks like chips, popcorn.
Margarine	Margarine
Olive oil	Olive oil
Sunflower oil	Sunflower oil
Mayonnaise and ketchup	Mayonnaise and mayonnaise-based products; ketchup
High-energy drinks	Sweetened drinks including sports drinks, bottled tea, syrup-based drinks
Low-energy drinks	Diet coke or diet sodas, flavored water included (no added sugar)
Coffee	Coffee
Tea, herbal tea	Tea, herbal tea
Wine	Red wine, white wine
Beer	Beer
Liquors	Liquors
Precooked food	Precooked food
Tofu	Tofu
