# Supplementary material for: Changes in Dietary Patterns through a Nutritional Intervention with a Traditional Atlantic Diet: The Galiat Randomized Controlled Trial
Source: Nutrients. 2021 Nov 25;13(12):4233. doi: 10.3390/nu13124233 (PMC8704078; doi:10.3390/nu13124233)
Supplement: Supplementary file 1 [file nutrients-13-04233-s001.zip › Supplementary Table S2.pdf]

Supplementary Table S2. Dietary patterns scores (with their 95% confidence intervals) identified by principal component analysis for intervention and control groups at baseline and after 6-months (per-protocol data set).

	**Intervention group**		**Control group**		**Adjusted mean differences**	**P-value**	**ICC**
	**Baseline**	**6 months**	**Baseline**	**6 months**			
Caloric	−0.131 (−0.307, 0.044)	−0.587 (−0.729, −0.446)	0.034 (−0.168, 0,.237)	−0,019 (−0.187, 0.149)	−0.443 (−0.657, −0.229)	<0.001	0.238
Frieds	−0.141 (−0.330, 0.047)	−0.632 (−0.785, −0.480)	0.125 (−0.054, 0.304)	−0.111 (−0.282, 0.060)	−0.374 (−0.603, −0.144)	0.001	0.328
Fruits, vegetables, and dairy products	0.165 (−0.008, 0.337)	0.468 (0.279, 0.656)	−0.132 (−0.294, 0.029)	−0.283 (−0.460, −0.105)	0.565 (0.319, 0.813)	<0.001	0.268
Alcohol	0.006 (−0.149, 0.160)	−0.275 (−0.385, −0.165)	−0.002 (−0.164, 0.159)	−0.211 (−0.356, −0.065)	−0.086 (−0.245, 0,073)	0.289	0.092
Fish and boiled meals	−0.021 (−0.174, 0.133)	0.276 (0.118, 0.433)	0.043 (−0.120, 0.206)	−0.178 (−0.331, −0.024)	0.481 (0.212, 0.749)	<0.001	0.426
