# Supplementary material for: Changes in Dietary Patterns through a Nutritional Intervention with a Traditional Atlantic Diet: The Galiat Randomized Controlled Trial
Source: Nutrients. 2021 Nov 25;13(12):4233. doi: 10.3390/nu13124233 (PMC8704078; doi:10.3390/nu13124233)
Supplement: Supplementary file 1 [file nutrients-13-04233-s001.zip › Supplementary Table S3.pdf]

Supplementary Table S3. Relation between baseline to 6-month mean changes in body weight (β coefficients) and baseline to 6-month mean changes in the dietary pattern scores using the entire sample independent of group allocation (per-protocol data set).

Change in food pattern score [1]	Coefficient (95% CI)	P
Caloric	0.172 (0.002, 0.343)	0.047
Frieds	0.250 (0.065, 0.434)	0.008
Fruits, vegetables, and dairy products	−0.186 (−0.375, 0.002)	0.053
Alcohol	0.025 (−0.177, 0.226)	0.881
Fish and boiled meals	−0.074 (−0.236., 0.089)	0.374

[1] Food pattern exposure variables are measured as change in factor score between baseline to 6-months. All food patterns are tested together in the same model. 95% CI, 95% confidence interval.
